# Association of Serum Vitamin D3 Levels With Vitiligo: A Case-Control Study at a Tertiary Care Center

**DOI:** 10.7759/cureus.87452

**Published:** 2025-07-07

**Authors:** Akkineni Usha Sri, Dilipchandra Chintada, Aruna Bathina, Kirankanth Vudayana, Mohammed Khatija Begum, Sravya Laveti

**Affiliations:** 1 Dermatology, Venereology, and Leprosy, Great Eastern Medical School and Hospital, Srikakulam, IND

**Keywords:** chemiluminescence method, melanocytes, tyrosinase activity, veti score, vitamin d deficiency, vitamin d levels and veti score, vitamin d sources, vitiligo

## Abstract

Introduction

Vitiligo is a skin condition that appears as white blotches and is brought on by the epidermis losing melanocytes, the cells that give skin its color. Numerous theories have been proposed to explain it, but the exact cause is still unclear. Vitamin D3, an essential vitamin, plays a role in immunological response and may help regulate melanocyte activity. Vitiligo and other autoimmune diseases have been linked to low levels of this powerful immune-modulating vitamin. Moreover, vitamin D3 promotes tyrosinase activity and melanin formation via binding to the vitamin D receptor in melanocytes. The primary objective of the study is to determine if the vitamin D levels are impaired and different in vitiligo patients compared to those in healthy individuals, and the secondary objective is to ascertain the impact of this impairment on the severity and scope of the illness.

Methodology

This study is a prospective case-control design. Over a 12-month period from March 2024 to March 2025, patients visiting the dermatology outpatient department (OPD) at our tertiary care hospital diagnosed with vitiligo were randomly enrolled as cases. Age- and gender-matched healthy individuals attending the hospital were recruited as controls. There were 40 vitiligo sufferers and 40 control subjects in the study, who were taken as per the cases visiting our OPD. The Vitiligo Extent Tensity Index (VETI) score was used to evaluate the degree of skin involvement in vitiligo patients. In order to evaluate serum vitamin D3 levels using the chemiluminescence method, blood samples were obtained from both cases and controls after informed consent and clearance from the institutional ethical committee. Based on their vitamin D3 levels, individuals were divided into three groups: inadequate (<20 IU), insufficient (20-30 IU), and normal (>30 IU). The mean and standard deviation were computed based on the results.

Results

The age and gender distribution of the vitiligo patients and the control group in this study did not differ significantly. The gender distribution of the 40 cases and 40 controls was equal, with 50% of the participants being men and 50% being women. The largest percentage of participants (37.5%) were between the ages of 41 and 60, followed by those between the ages of 21 and 40 (35%), 11 and 20 (17.5%), one and 10 (10%). Vitiligo patients had a mean serum vitamin D level of 25.1 ± 10.6 ng/mL, while the control group had a significantly higher level of 37.9 ± 26.0 ng/mL (t value = -2.88 and p = 0.0057, statistically significant). Furthermore, chi-square value = 10.60 and p = 0.0314 indicated a statistically significant correlation between serum vitamin D levels and the degree of vitiligo as determined by the VETI score. The study showed that vitiligo patients had lower vitamin D levels compared to those of healthy subjects, and it also made clear how vitamin D deficiency may affect the severity and extent of vitiligo.

Conclusion

Vitiligo is recognized as a complex disorder with multiple factors. Melanocytes possess vitamin D receptors, which indicates that vitamin D might be involved in regulating their function. Thus, we investigated the role of vitamin D in the etiopathogenesis of vitiligo. The study indicates an inverse correlation of serum vitamin D3 levels with the degree of vitiligo.

## Introduction

Vitiligo is an acquired, typically progressive skin disorder marked by the loss of melanocytes, leading to well-defined areas of depigmentation that vary in shape and size [1]. It is considered a chronic autoimmune condition in which the disease process is initiated by dysfunctional melanocytes that activate immune responses, ultimately leading to epidermal melanocytes being destroyed. The fundamental mechanisms have been explained by a number of concepts, including the "double-hit" theory, autoimmune cytotoxicity, oxidative stress, biochemical imbalances, the melanocytorrhagy hypothesis, and reduced melanocyte survival. Those who have a family history of vitiligo or who suffer from other autoimmune conditions such as thyroid issues, Addison's disease, pernicious anemia, type 1 diabetes mellitus, psoriasis, or rheumatoid arthritis are more likely to acquire vitiligo [2]. Vitiligo is a common dermatological condition worldwide, affecting both men and women, though it is slightly more prevalent among women. Notably, more than 60% of individuals develop symptoms before reaching 30 years of age [3].

Ultraviolet B (UVB) rays cause a photochemical reaction in the skin that results in the production of vitamin D, an essential hormone. 7-Dehydrocholesterol in the skin is changed by UVB radiation into pre-vitamin D3, which then goes through thermal isomerization to become vitamin D3, particularly during the summer months when UVB exposure is greater. However, several factors can limit cutaneous vitamin D synthesis, including increasing age, higher levels of skin pigmentation, the use of sunscreen, and clothing that covers most of the skin surface [4].

Ergocalciferol, often known as vitamin D2, and cholecalciferol, commonly referred to as vitamin D3, are the two primary forms of vitamin D. Both of them can be acquired through dietary sources. While vitamin D3 is mostly found in animal goods, especially fatty fish, vitamin D2 is mostly found in plant-based sources such as fungus and yeast. Fortified milk, cheese, eggs, and cereals are additional dietary sources of vitamin D [5].

Multiple studies have indicated that individuals with vitiligo are more prone to vitamin D deficiency compared to the general population. This is likely due in part to reduced sun exposure, as many vitiligo patients avoid sunlight to minimize further depigmentation, which in turn restricts the natural production of vitamin D. Such deficiency may aggravate immune system imbalances, potentially playing a role in the progression of vitiligo [6]. Melanocytes have been found to possess vitamin D receptors, suggesting that vitamin D supplementation may help preserve their function and viability. This could promote melanin production and support repigmentation in those affected by vitiligo. Additionally, the antioxidant effects of vitamin D may help mitigate oxidative stress, an important factor in vitiligo development, thereby potentially slowing or preventing further disease progression [7].

## Materials and methods

Study structure

Over the course of 12 months, from March 2024 to March 2025, this prospective case-control study was carried out in the dermatology department of Great Eastern Medical School and Hospital (GEMS), Srikakulam, Andhra Pradesh, India. The Institutional Ethics Committee of GEMS granted ethical approval for the study (approval number: 179/IEC/GEMS&H/2024).

Study cohort

Forty patients with vitiligo (generalized, focal, or segmental) of all ages and sexes who visited our tertiary care dermatology outpatient department (OPD) were included in the study. They were chosen at random from the dermatology OPD register using a computer-generated random number table. For every chosen case, a healthy control who was matched by age (±1 year) and gender was gathered among patients receiving general outpatient care, making sure they fulfilled the inclusion and exclusion criteria (40 controls). Exclusion criteria included pregnant or lactating women, individuals with a history of autoimmune diseases, controls with a past history of autoimmune conditions, and those who had used vitamin D supplements. Furthermore, vitiligo patients who had undergone phototherapy or taken vitamin D supplements for any reason in the past year were also excluded from the study.

Data collection

Patients who came to our outpatient department (OPD) with depigmented skin lesions gave their consent and information about their age, gender, occupation, socioeconomic position, history (complaints, onset, and progression), cutaneous examination, and other facts. The Vitiligo Extent Tensity Index (VETI) score was used to quantify the affected body surface area without blinding, and clinical photos were collected to assess the severity of the disorder [8]. This scoring was based on standardized clinical criteria and photographic documentation to maintain objectivity and reproducibility. A single experienced dermatologist evaluated it to ensure consistency and eliminate inter-observer variability. The VETI evaluates the degree of vitiligo by calculating a score that takes into account both the severity and the expanse of the disorder, guaranteeing a reliable and consistent outcome. The "rule of nines," which is frequently applied in burn assessments, was utilized to determine the percentage of skin involvement (p). Five phases of illness intensity (T) were used to score the afflicted parts, which included the head (h), upper limbs (u), trunk (t), lower limbs (l), and genitalia (g): Stage 0, skin that is normal; Stage 1, hypopigmentation, which includes lighter uniform pigmentation and trichrome; Stage 2, perifollicular pigmentation and total depigmentation with black hair; Stage 3, no perifollicular pigmentation and total depigmentation with black hair; Stage 4, complete depigmentation with black and white hair, with or without perifollicular pigmentation; and Stage 5, significant hair lightening and total depigmentation. Because of their extreme resistance to treatment, areas such as the extremities, where hair does not normally grow, are likewise regarded as grade 5.

A formula that considers the contributions from all impacted bodily regions is used to determine the body's overall VETI score: (head involvement percentage × severity grade) + (trunk involvement percentage × severity grade) × 4 + (upper limb involvement percentage × severity grade) × 2 + (lower limb involvement percentage × severity grade) × 4 + (genital involvement percentage × severity grade) × 0.1.

According to the "rule of nines," the coefficients in this formula represent the percentage of skin surface area. Therefore, the coefficient is 1 (9:9 = 1) for the head, 4 (36:9 = 4) for the trunk and lower limbs, 2 (18:9 = 2) for the upper limbs, and around 0.1 (1:9 = 0.1) for the genitalia, where the proportion of being involved is p and tensity is T.

VETI = (Ph × Th) + (Pt × Tt) × 4 + (Pu × Tu) × 2 + (Pl × Tl) × 4 + (Pg × Tg) × 0.1 is the formula's representation. For complete body involvement, the score would be 5 + 20 + 10 + 20 + 0.5 = 55.5. Therefore, the maximum possible VETI score is 55.5.

Following ethics committee permission and informed consent, blood samples were drawn from the patients and controls in order to use the chemiluminescence method to evaluate serum vitamin D3 levels. Although formal blinding was not implemented due to the clinical setting, the observer was not involved in the biochemical analysis or statistical interpretation of vitamin D levels. This separation of roles helped reduce confirmation and observer bias.

Chemiluminescence method

This method involves conjugating vitamin D to an isoluminol derivative and coating magnetic particles (solid phase) with a particular antibody that targets vitamin D. 25-Hydroxyvitamin D (25(OH)D) separates from its binding protein during incubation and vies with the labeled vitamin D for antibody binding sites. After incubation, a wash cycle is used to get rid of any loose material. A flash chemiluminescent reaction is then started by adding the beginning reagents. A photomultiplier detects the ensuing light signal, whose strength is inversely proportional to the samples' 25-hydroxyvitamin D concentration [9]. The mean and standard deviation of vitamin D levels were calculated based on the obtained results. The sensitivity of this method is 93%-95%.

The subjects were then classified into four categories based on the levels of vitamin D3 in serum (Table 1).

**Table 1:** Interpretation of vitamin D level

Level	Reference Range
Deficient	<20 ng/mL
Insufficient	20-30 ng/mL
Normal/Sufficient	31-70 ng/mL
Toxic	>100 ng/mL

Statistical analysis

A software for statistical analysis, SPSS version 25 (IBM Corp., Armonk, NY), was used. The p-value was deemed statistically significant if it was less than 0.05.

## Results

Demographic data of the vitiligo and control groups

Age

Overall, the data indicates that cases are more common in the age group of 41-60 years, which accounted for 37.5%, followed by 21-40 years at 35%, 11-20 years at 17.5%, and 1-10 years at 10% (Table 2).

**Table 2:** Distribution of participants by age

Age (Years)	Cases	Controls
1-10	4 (10%)	1 (2.5%)
11-20	7 (17.5%)	8 (20%)
21-40	14 (35%)	15 (37.5%)
41-60	15 (37.5%)	16 (40%)
>60	0	0

Gender

In the study, 50% of the 40 cases were men, and 50% were women, while in the control group of 40 individuals, 50% were men, and 50% were women.

Duration

Forty-five percent of the cases were suffering with vitiligo for >2 years, and 35% were suffering with vitiligo for <1 year, and 20% were suffering for 1-2 years.

Type

**Figure 1:**
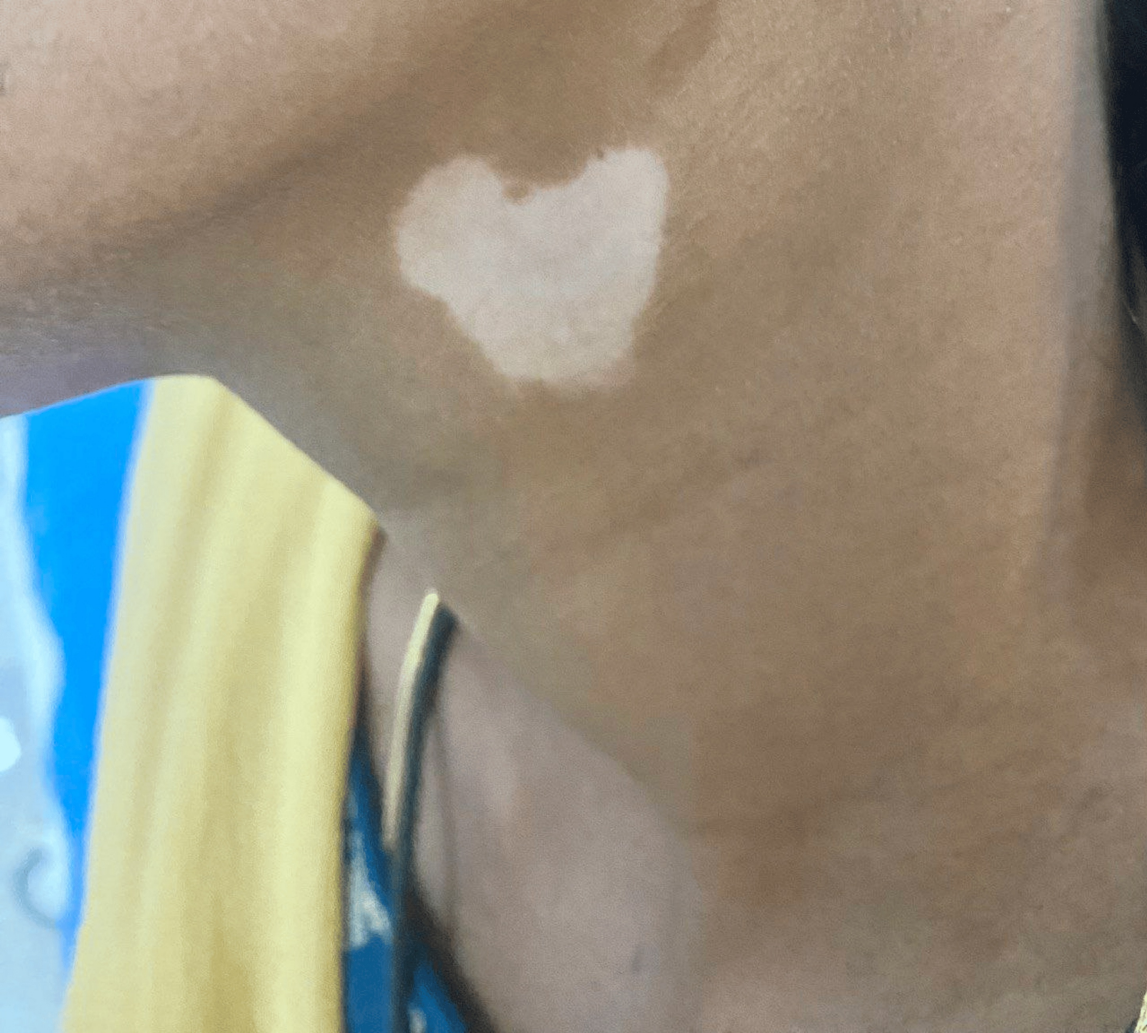
Segmental vitiligo

**Figure 2:**
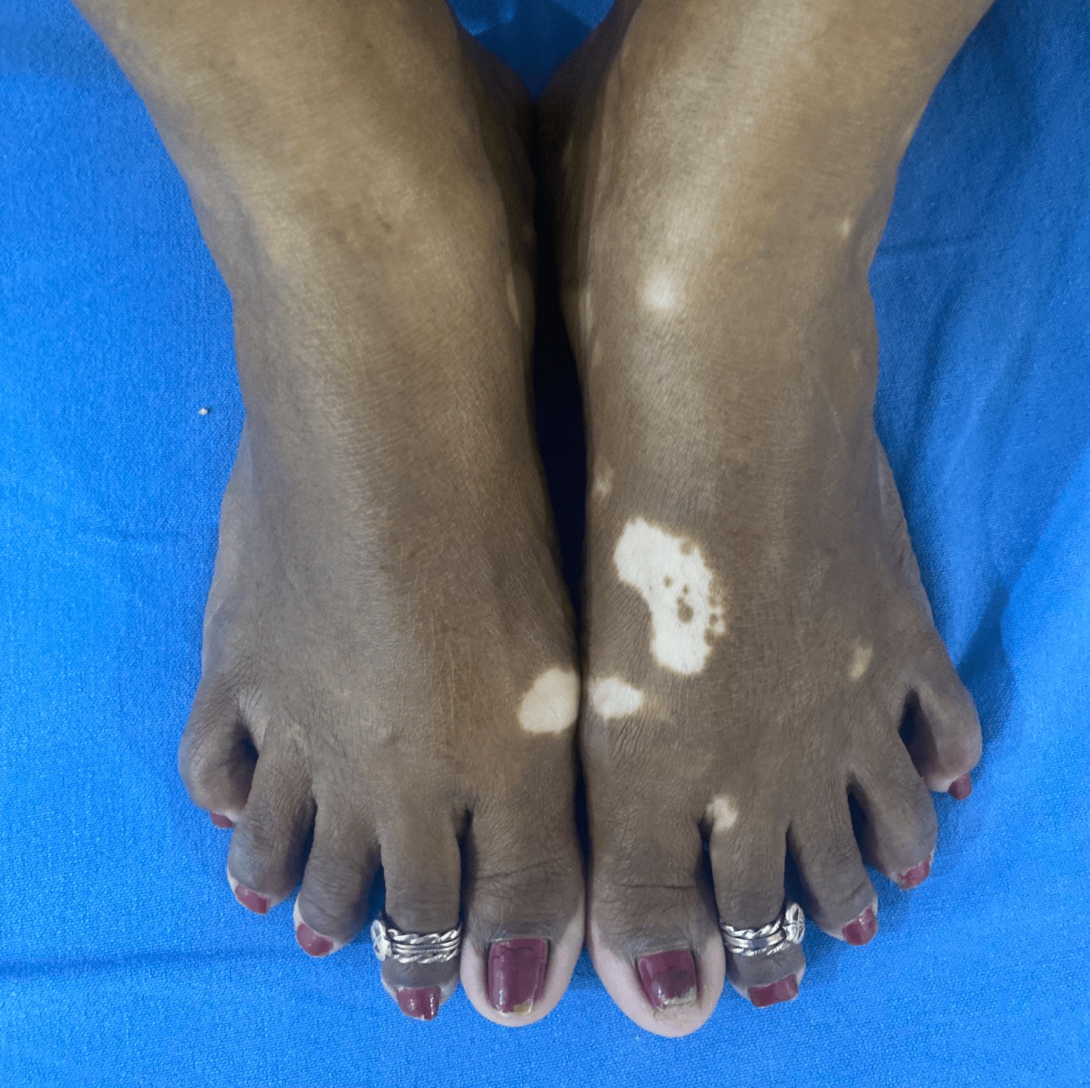
Non-segmental vitiligo

**Figure 3:**
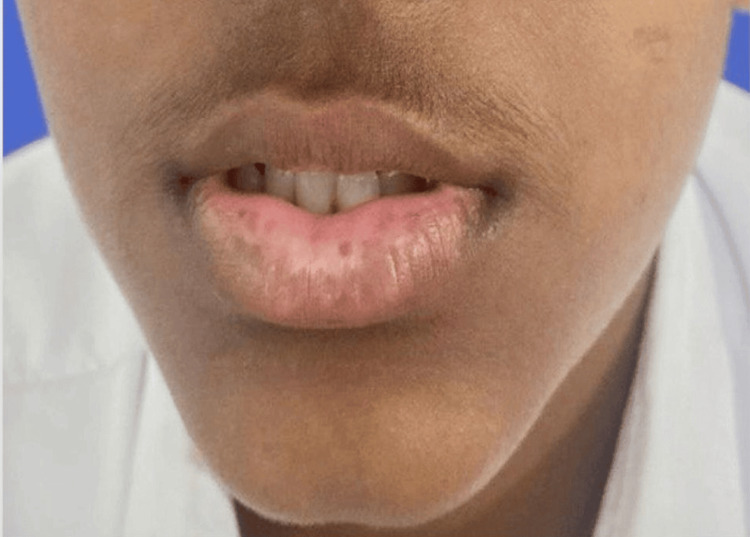
Lip vitiligo

**Figure 4:**
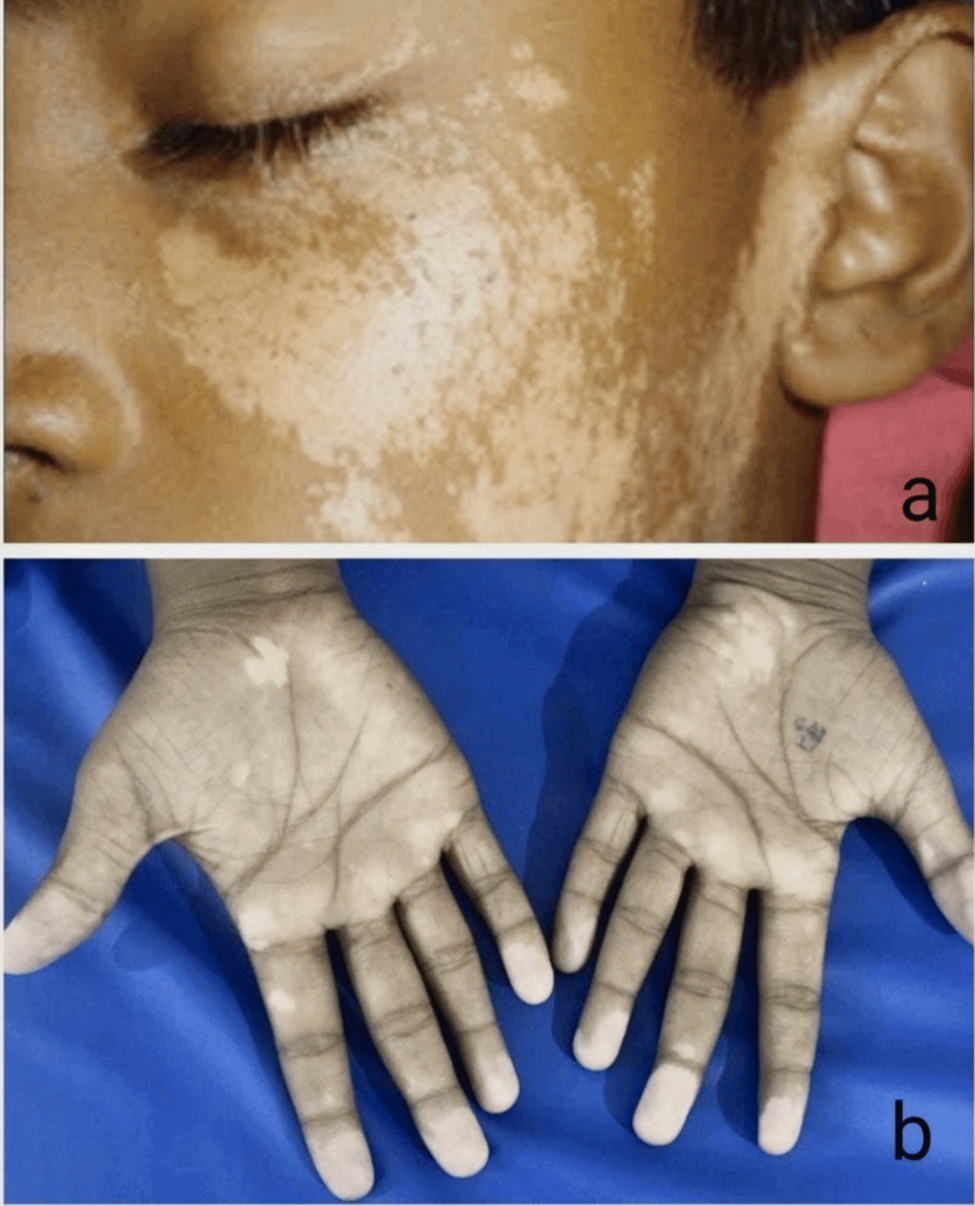
Acrofacial vitiligo (a) Face lesions and (b) hand lesions

Of the 40 cases in this study, 10 cases (25%) had segmental vitiligo, which can be unilateral or bilateral and confined to a specific segment (Figure 1); 23 cases (57.5%) had non-segmental vitiligo, which involves depigmented macules and patches on both sides of the body (Figure 2); six cases (15%) had lip vitiligo, which is characterized by depigmented macules and patches confined to the lips (Figure 3); and one case (2.5%) of acrofacial vitiligo presented with depigmented macules and patches involving the face, hands, and feet (Figure 4).

VETI Score

Most of the cases have a VETI score of >10, i.e., 55%, followed by a VETI score of 5-10, i.e., 25%, and a VETI score of <5, i.e., 20% (Table 3).

**Table 3:** Distribution of patients according to VETI score VETI: Vitiligo Extent Tensity Index

VETI Score	Number of Cases
<5	8 (20%)
5-10	10 (25%)
>10	22 (55%)

Vitamin D Levels

The vitamin D levels of all the cases and controls were measured using the chemiluminescence method, and the data was compared (Table 4). Later, the association between vitamin D levels and VETI score was measured, and the relationship between vitamin D and the development and spread of vitiligo lesions was shown to be statistically significant (Table 5).

**Table 4:** Comparison of vitamin D levels in vitiligo patients and control subjects t value = -2.88; p = 0.0057 (statistically significant); N = number; % = proportion SD: standard deviation

Vitamin D Levels (ng/mL)	Cases (N)	Controls (N)	P-value
Deficient	16 (40%)	10 (25%)	
Insufficient	14 (35%)	9 (28%)	
Normal/Sufficient	10 (25%)	19 (47.5%)	
Toxicity	0 (0)	1 (2.5%)	
Total	40	40	
Mean + SD	25.1 ± 10.6	37.9 ± 26.0	0.0057

**Table 5:** Association between vitamin D levels and VETI score Chi-square value = 10.60; p = 0.0314 VETI: Vitiligo Extent Tensity Index

VETI Score	Deficient	Insufficient	Sufficient	P-value
<5	2 (5%)	2 (5%)	6 (15%)	
5-10	4 (10%)	4 (10%)	3 (7.5%)	
>10	10 (25%)	8 (20%)	1 (2.5%)	
Total	16 (40%)	14 (35%)	10 (25%)	0.0314

## Discussion

The control group's mean age was 36.31 ± 4.8 years, whereas the case group's mean age was 33.5 ± 16.8 years. Regarding gender and age, there was no statistically significant difference between the two groups.

Vitiligo patients in this study had an average vitamin D level of 25.1 ± 10.6 ng/mL, while the control group had an average of 37.9 ± 26.0 ng/mL. This suggests that the vitiligo patients had significantly lower vitamin D levels than healthy controls. With t value = -2.88 and p = 0.0057, the difference was statistically significant.

These results are in line with research by Abdul-Reda and Al-Zobaidy, who found no discernible gender differences between the groups, including 50 vitiligo patients (24 men and 26 women) and 50 healthy control subjects in their study [10]. The mean serum vitamin D levels in the vitiligo group were 15.08 ± 8.29 ng/mL and 20.27 ± 9.05 ng/mL in the control group, with a statistically significant difference of 0.05.

In another study by Mahmmod and Ismael (2021) that included 46 vitiligo patients, the majority of vitiligo patients had significantly lower vitamin D levels than in the control group. This difference was statistically significant (p < 0.05). However, no significant difference was observed in terms of age and gender, with the mean age of vitiligo patients being 24.24 ± 12.28 years and 29.21 ± 13 years in the control subjects, which is consistent with our findings [11].

A study by Farag et al. included 50 patients with different degrees of vitiligo severity, along with 25 age-matched, sex-matched, and skin phenotype-matched controls [12]. Patients with vitiligo were treated with narrowband UVB (NBUVB) thrice weekly for 12 weeks. Baseline serum 25(OH)D levels by enzyme-linked immunosorbent assay (ELISA) and vitiligo area severity index (VASI) were estimated and then reevaluated after NBUVB sessions, which demonstrated a statistically significant difference in baseline serum 25(OH)D levels between vitiligo patients and controls, with p < 0.001.

Several studies, including those by Ustun et al. [13] and Hassan et al. (2019) [14], included 80 vitiligo patients and 20 age- and sex-matched controls. Patients with vitiligo received NBUVB treatment biweekly for a duration of 24 weeks, and 25-hydroxyvitamin D levels were measured at zero, 12, and 24 weeks in the cases and at zero in controls by ELISA, and vitiligo area severity index (VASI) calculated at 0 and 24 weeks showed that while the mean vitamin D levels in vitiligo patients were lower than in controls, this difference was not statistically significant. Nevertheless, we found that this difference was statistically significant in our investigation.

With chi-square value = 10.60 and p = 0.0314, this study found a statistically significant correlation between vitamin D levels and the presence and severity of vitiligo lesions as determined by the VETI score.

Previous studies by Mahmmod and Ismael (2021) [11], Hassan et al. [14], and Saleh et al. [15] also investigated the correlation between vitamin D levels and VETI score, but they found no statistically significant difference. In contrast, our study identified a significant association. Additional research with bigger sample sizes and longer follow-up times is required to fully comprehend this association.

Vitamin D levels in serum and tissue are believed to affect pigmentation through the expression of the *VDR* gene because vitamin D stimulates melanocyte activity and melanogenesis. Consequently, there is an inverse association between vitamin D levels and the VETI score.

For instance, research has shown that people with more extensive vitiligo typically have lower amounts of vitamin D in their blood. This deficiency may worsen the autoimmune processes driving the disease, implying that maintaining sufficient vitamin D levels could be beneficial in managing vitiligo [16].

Vitamin D supplementation has demonstrated encouraging results in vitiligo sufferers. Topical vitamin D3 analogues have emerged as a new therapeutic option in the treatment of vitiligo [16].

Several studies have since reported the use of vitamin D analogues, either alone or in combination with ultraviolet light or corticosteroids, to promote repigmentation in vitiligo treatment [17].

Vitamin D3 analogues, whether used alone or in combination with other therapies, may aid in repigmentation in vitiligo patients [18]. Multiple studies have shown that vitamin D3 promotes repigmentation in vitiligo lesions by enhancing tyrosinase activity and stimulating melanogenesis, as observed in various in vitro experiments [19].

Various parameters such as skin type, geographic location, and sun exposure can influence vitamin D levels, which may complicate the interpretation of results and leads to inconsistent findings. The cost and accessibility of the test are other limitations.

## Conclusions

Vitiligo patients' average vitamin D levels were considerably lower than those of the control group. Furthermore, there was a statistically significant inverse correlation found between vitamin D insufficiency and illness severity as determined by the VETI score. These results underline the necessity of further studies regarding the benefits of vitamin D supplementation in vitiligo patients. Due to its immunomodulatory and melanogenesis-related functions, vitamin D insufficiency affects the severity of vitiligo and may be a contributory factor to its development. Thus, evaluating serum vitamin D levels in vitiligo patients helps in better treatment outcomes.
